# Knowledge, attitudes, and behaviors of practitioners supporting cancer patients in fertility preservation in DKI Jakarta: A cross-sectional study

**DOI:** 10.18502/ijrm.v20i2.10506

**Published:** 2022-03-21

**Authors:** Achmad Kemal Harzif, Raymond Surya, Raden Muharam, Gita Pratama, Alfa Putri Meutia

**Affiliations:** ^1^Division of Immuno-Endocrinology and Fertility, Department of Obstetrics and Gynecology, Faculty of Medicine, Universitas Indonesia, Dr. Cipto Mangunkusumo Hospital, Jakarta, DKI Jakarta Province, Indonesia.; ^2^Department of Obstetrics and Gynecology, Faculty of Medicine, Universitas Indonesia, Dr. Cipto Mangunkusumo Hospital, Jakarta, DKI Jakarta Province, Indonesia.; ^3^Division of Reconstruction Urogynecology, Department of Obstetrics and Gynecology, Faculty of Medicine, Universitas Indonesia, Dr. Cipto Mangunkusumo Hospital, Jakarta, DKI Jakarta Province, Indonesia.

**Keywords:** Knowledge, Attitude, Practice, Doctor, Fertility preservation.

## Abstract

**Background:**

More than 135,000 people aged under 45 yr are diagnosed with cancer annually in Indonesia. Good detection and management of cancer increase the quality of life.

**Objective:**

To determine the knowledge, attitudes, and behaviors of practitioners supporting cancer patients in fertility preservation.

**Materials and Methods:**

This cross-sectional study was conducted in 18 type D government hospitals and Dr. Cipto Mangunkusumo Hospital, in Jakarta, Indonesia, between January 2018 and August 2019. This study involved practitioners providing care to cancer patients. Data were described descriptively.

**Results:**

Most of the general practitioners, specialists, and subspecialists who participated in this study were aged 26-30 yr (65.4%), 31-35 yr (70.4%), and 31-40 yr (53.0%), respectively. The fertility treatment most known by general practitioners was in vitro fertilization with embryo cryopreservation (12.1%); for specialists it was sperm cryopreservation (24.5%). Meanwhile, subspecialists knew most about in vitro fertilization with embryo cryopreservation and sperm cryopreservation using a GnRH agonist (such as leuprolide injection) pre-cancer treatment (13%). A positive attitude towards fertility preservation as an important priority for cancer patients was shown in 72.0% of general practitioners, 73.3% of specialists, and 100% of subspecialists. General practitioners mostly referred patients to fertility specialists (44.4%). Many specialists (54.9%) and subspecialists (67%) discussed the possible impact of the patient's condition and / or treatment on fertility.

**Conclusion:**

The knowledge of and practices related to fertility preservation differed among general practitioners, specialists, and subspecialists. However, positive attitudes among them were similar.

## 1. Introduction

In Indonesia, based on Riset Kesehatan Dasar 2013*, *the prevalence of cervical and breast cancer were 0.08% (98,692 people) and 0.05% (61,682 people) in 2012, respectively. There are more than 135,000 people below 45 yr old who are diagnosed with cancer every year in Indonesia (1). The advancement of technology to screen and treat cancer has increased the survival rate of cancer patients. Unfortunately, long-term cancer therapy, such as chemotherapy and radiotherapy, can have negative psychological, economic, social, sexual, and biological effects (2). The National Comprehensive Cancer Network in 2014 stated that one of the most important concerns among young cancer patients was an option for fertility preservation (3, 4). One study found that few patients in the study were counselled about fertility preservation. This was due to a lack of knowledge on the optimal time and methods to preserve fertility (5).

The profile of knowledge, attitudes, and practices among specialists treating cancer patients is still varied. Several studies such as those conducted by Adams and colleagues (6) in the UK and Overbeek et al. (7) in the Netherlands have shown that oncologists recognize that fertility preservation is an important issue. However, less than 50% of oncologists in these studies discussed the choices of patients due to lack of time, lack of knowledge, bad patient prognosis, and fertility treatment variable success rate and unaffordable cost. Lack of health providers' knowledge will influence their attitudes and practices.

No studies have been carried out about health care providers' knowledge, attitudes, and practices surrounding fertility preservation in cancer patients; therefore, this study aimed to determine the profile of these, focusing on Jakarta, the capital city of Indonesia.

## 2. Materials and Methods 

### Participants

This descriptive study with a cross-sectional design was conducted in all type D government hospitals with general practitioners and specialists in Jakarta, and in Dr. Cipto Mangunkusumo Hospital in Jakarta with subspecialists, from January 2018 to August 2019. The participants consisted of general practitioners, pediatricians, subspecialists in hematology-oncology, interns, intern subspecialists in hematology-oncology, general surgeons, oncology surgeons, and radiotherapy specialists.

### Questionnaire

The questionnaire was adapted from a questionnaire by the British Journal of Cancer from 2013 with the title fertility preservation in cancer survivors: A national survey of oncologists' current knowledge, practice, and attitudes. The results of its translation and validation in the Indonesian language have been published by Harzif et al. (8).

### Ethical considerations

This study was approved by the Ethics Committee of the Faculty of Medicine, University of Indonesia (Code: 926/UN2.F1/ETIK/2017). Written informed consent was obtained from all participants.

### Statistical analysis

The data gathered from the completed questionnaires were described by frequency and percentage. The analysis used the Statistical Package for the Social Sciences (SPSS), version 23.0.

## 3. Results

Within the 18 type D government hospitals in Jakarta, 133 of the 215 general practitioners (61.9%) fully completed the questionnaire, as did 21 of 40 (52.5%) internists, 18 of 41 (43.9%) pediatricians, 12 of 25 (48.0%) general surgeons, and 20 of 39 (51.3%) obstetricians and gynecologists. In Dr. Cipto Mangunkusumo Hospital in Jakarta, two of the 13 intern subspecialists in hematology-oncology (15%) fully completed the questionnaire, as well as five of the 18 (63%) pediatrician subspecialists in hematology-oncology, two of the six (33%) oncologist surgeons, and six of the 10 (60%) radiotherapy specialists.

Around 65.4% of the general practitioners were 26-30 yr old and 46.6% had graduated from Government University. 40.6% were members of Java tribes. Among the specialists, 70.4% were 31-35 yr old and 60.6% had graduated from Universitas Indonesia. Meanwhile, all of the subspecialists had graduated from Universitas Indonesia with an average age of 31-40 yr (53%).

The general practitioners, specialists, and subspecialists most knew about in vitro fertilization (IVF) with embryo cryopreservation (12.1%), sperm cryopreservation (25.4%), and IVF with embryo preservation and sperm cryopreservation using a GnRH agonist pre-cancer treatment (13%), respectively. A positive attitude about fertility preservation as an important priority for cancer patients was shown among 72.0% of general practitioners, 73.3% of specialists, and 100% of subspecialists. The general practitioners often referred patients who had questions about fertility to a fertility specialist (44.4%). The specialists and subspecialists mostly discussed the impact that a patient's condition and/or treatment might have on their future fertility (54.9% and 67%, respectively). Table I describes the factors influencing health care providers when initiating a discussion about fertility preservation. Table II depicts comments from health care providers about fertility preservation.

**Table 1:** Factors that health care providers consider when deciding whether to initiate a discussion about fertility preservation


**Factors**		**GP **	**S **	**SS **
**Poor success rates of fertility preservation options **		110 (82.7)	61 (85.9)	9 (60)
**Lack of fertility services in the area **		115 (86.5)	61 (85.9)	11 (73)
**Constraints on provider's time **		97 (72.9)	49 (69.0)	8 (54)
**Limited knowledge of fertility preservation options **		127 (95.5)	59 (83.1)	13 (87)
**Burden to patients **		118 (88.7)	64 (90.2)	11 (74)
**Someone else within provider's practice discusses fertility preservation with patients**		96 (72.2)	50 (70.5)	7 (47)
**The patient**				
	**Is too ill to delay treatment to pursue fertility preservation **	124 (93.2)	67 (94.4)	12 (80)
	**Cannot afford fertility preservation **	123 (92.5)	65 (91.6)	11 (73)
	**Has a hormonally-sensitive malignancy **	124 (93.2)	69 (97.2)	11 (73)
	**Does not want to discuss fertility preservation **	124 (93.3)	68 (95.8)	11 (73)
	**Has a poor prognosis **	124 (93.2)	69 (97.2)	12 (80)
	**Is single **	121 (91.0)	66 (93.0)	12 (80)
	**Already has a child or children **	127 (95.5)	66 (93.0)	12 (80)
Data shown as n (%). GP: General practitioners, S: Specialists, SS: Subspecialists				

**Table 2:** Comments of health care providers about fertility issue


**Comments**		**GP **	**S **	**SS **
**Need further information about fertility preservation **		121 (91.0)	53 (74.6)	14 (93)
**Limit for preservation in women (40 yr old)**		76 (57.1)	52 (73.2)	6 (40)
**Limit for preservation in men (never)**		52 (39.1)	46 (64.8)	9 (60)
**Guidelines for fertility preservation (never read)**		84 (63.2)	53 (74.6)	7 (47)
**Relationship with fertility specialists (good)**		47 (35.3)	35 (49.3)	9 (60)
**Distance to fertility preservation referral (same city)**		98 (73.7)	53 (74.6)	1 (7)
**Characteristics of patients prioritized for fertility preservation**				
	**Women**	58 (43.6)	38 (53.5)	8 (53)
	**Higher economic level**	106 (79.7)	46 (64.8)	8 (53)
	**Bachelor**	84 (63.2)	48 (67.6)	6(40)
Data shown as n (%). GP: General practitioners, S: Specialists, SS: Subspecialists				

## 4. Discussion

Most of the general practitioners had never had patients that were using fertility preservation methods and only a few knew about the choices concerning them. A study conducted in Indonesia by Harzif et al. (9) stated that this is common due to a lack of specialized organizations or local guidance concerning fertility preservation. This lack of general practitioners' knowledge in Indonesia is also seen in the United States where less than 25% of general practitioners participating in one study could present educational material about fertility preservation (10). Around 91% of them needed further information about fertility preservation; only a few had discussed this topic with fertility specialists, given written information about fertility preservation, or considered patients' hopes about their future fertility. This may be because of a lack of knowledge in fertility preservation, newly started fertility preservation facilities in Indonesia, unclear referral schematics, and unaffordable costs which have not been supported by universal health coverage.

Among specialists, the response rate was from 43.9% to 52.5%. This response rate was better compared with a study in Hong Kong which had a response rate of 36.5% (11). The three most known methods in our study's specialists were IVF with embryo cryopreservation, sperm cryopreservation, and methods carried out pre-cancer treatment using a GnRH agonist. In the Hong Kong study, the three most familiar fertility preservations were similar: sperm cryopreservation, oocyte cryopreservation, and IVF with embryo cryopreservation (11).

Around 74.6% of specialists and 93.0% of subspecialists still needed further information about fertility preservation. In a study conducted in Lebanon, 89% of oncologists agreed that cancer treatment can threaten fertility and 94.4% agreed to discuss fertility issues with patients. Specialists and subspecialists often discussed the impact of a patient's condition on fertility in the future (12). Quinn and colleagues showed that oncologists in their study were 4.9 times more likely to discuss about the influence of cancer treatment on fertility. Meanwhile, oncologist gynecologists or oncologist hematologists were 2.1 times more comfortable in discussing the option of fertility preservation with patients. However, less than 25% referred patients to fertility specialists. This was because fertility preservation services were only newly developed and there was a lack of informational media about these services (10). Other studies have shown that only 47% of oncologists in the United States and less than 20% in Saudi Arabia referred cancer patients to fertility centers (13, 14).

Factors influencing the discussion about fertility preservation included a bad prognosis, a patient being too ill to delay treatment, and being unable to afford to pay for the service. These factors were in accordance with another study which found that challenges in referring to fertility specialists included a high rate of recurrence and a bad prognosis (15-17). Oncologists also said that there was not enough time to perform fertility preservation activities before cancer treatment. Sperm preservation has a higher success rate than ovary preservation (18); therefore, this information should be used in counseling patients. Oncologists believed that the desire to refer depended on the attitudes about fertility preservation. In the Lebanese study, 90% of the oncologists had more than six yr experience; however, the knowledge of choices, success rates, and costs of fertility preservation methods was still limited. Therefore, this issue should be included in the curriculum of oncology training (12).

### Limitations

This study had the limitation of non-response bias due to potential differences in those who did not return a complete questionnaire. Apart from that, the data were based on questionnaire choices, so it was impossible for in-depth data to be collected and there was a risk for respondents misunderstanding the instructions.

## 5. Conclusion

The knowledge of and practices regarding fertility preservation were different among general practitioners, specialists, and subspecialists. Positive attitudes among them were similar.

##  Conflict of Interest

The authors declare that there is no conflict of interest.
